# New Canadian natural health product regulations: a qualitative study of how CAM practitioners perceive they will be impacted

**DOI:** 10.1186/1472-6882-6-18

**Published:** 2006-05-10

**Authors:** Karen Moss, Heather Boon, Peri Ballantyne, Natasha Kachan

**Affiliations:** 1 Association of Ontario Health Centres, 1 Eva Road, Etobicoke, ON, M9C 4Z5, Canada; 2 Leslie Dan Faculty of Pharmacy, University of Toronto, 19 Russell Street, Toronto, ON M5S 2S2, Canada

## Abstract

**Background:**

New Canadian policy to regulate natural health products (NHPs), such as herbs and vitamins were implemented on January 1^st^, 2004. We explored complementary and alternative medicine (CAM) practitioners' perceptions of how the new regulations may affect their practices and relationships with patients/consumers.

**Methods:**

This was an applied ethnographic study. Data were collected in fall 2004 via qualitative interviews with 37 Canadian leaders of four CAM groups that use natural products as a core part of their practises: naturopathic medicine, traditional Chinese medicine (TCM), homeopathic medicine and Western herbalism. All interviews were transcribed verbatim and coded independently by a minimum of two investigators using content analysis.

**Results:**

Three key findings emerged from the data: 1) all CAM leaders were concerned with issues of their own access to NHPs; 2) all the CAM leaders, except for the homeopathic leaders, specifically indicated a desire to have a restricted schedule of NHPs; and 3) only naturopathic leaders were concerned the NHP regulations could potentially endanger patients if they self-medicate incorrectly.

**Conclusion:**

Naturopaths, TCM practitioners, homeopaths, and Western herbalists were all concerned about how the new NHP regulations will affect their access to the products they need to practice effectively. Additional research will need to focus on what impacts actually occur as the regulations are implemented more fully.

## Background

Studies in Canada, the United States and the United Kingdom indicate that people who use natural health products (NHPs) like herbal medicines are more likely to do so as a component of self-care than upon the recommendation of a complementary/alternative medicine (CAM) practitioner [1-3]. However, in 2003, almost seven percent of Canadians reported seeing some type of CAM practitioner (chiropractor, massage therapist, acupuncturist, homeopath, naturopath, herbalist or other)[4]. When individuals do visit CAM practitioners, they are often advised about the use of NHPs for their particular conditions or problems, and NHPs are an important aspect of many CAM practitioners' practices.

Recently, the regulation of NHPs in Canada has changed; from previous status as foods with relatively unencumbered access by those who use NHPs as part of a therapeutic regime, to the current status where these products are regulated as a special category of drugs, with more strict control over their manufacturing, labelling, and indications for use. As part of the Health Products and Food Branch of Health Canada, the Natural Health Products Directorate (NHPD) is the regulating authority for NHPs for sale in Canada. The NHPD's role is "to ensure that Canadians have ready access to NHPs that are safe, effective and of high quality, while respecting freedom of choice and philosophical and cultural diversity" [5]. Although the new regulations do not directly impact the *practice *of health care practitioners (some of whom are unregulated, and some whose regulations fall under provincial jurisdiction), practitioners who use NHPs as part of their practice may be indirectly affected. We explore CAM practitioners' (naturopaths, traditional Chinese medicine (TCM) practitioners, Western herbalists and homeopaths) perceptions of how the new Natural Health Product Regulations (referred to in this paper as NHP regulations) may affect their practices. We also analyze the commonalties and differences in perceptions between these CAM groups' perceptions of the impact of the NHP regulations.

### The NHP regulations

In Canada, as of January 1^st^, 2004 the federal government regulates NHPs, such as herbs, vitamins and minerals. Under the new NHP regulations, by definition, a NHP must be safe for over-the-counter (OTC) use; that is, the label and package insert must provide enough information for a consumer to safely and effectively use the product without consulting a health care provider [6]. NHPs are defined in the regulations as herbs, vitamins, minerals, essential fatty acids and homeopathics which are used to prevent, diagnose or treat disease, restore or correct function or maintain or promote health that are endorsed for self-care purposes [6].

The new regulations stipulate that all NHPs sold in Canada require product licenses. The intent of requiring pre-market approval is to provide a mechanism for assessing and managing the benefits and risks associated with the use of NHPs [7,8]. The regulations set out the requirements for submitting an application for a product license, which includes the quantity of the medical ingredients, the purpose for which the NHP is intended to be sold as well as safety and efficacy data that supports the product [9]. Product license submissions must provide a comprehensive review of the evidence for the safety and efficacy of the product including evidence of traditional uses where appropriate [10]. Since up to 50,000 NHPs were already being sold in Canada when the regulations became law on January 1, 2004, Health Canada has provided a six year transition period during which companies must submit product license applications for all existing products. Products without licenses may continue to be sold during the transition period, but enforcement actions [9] will be taken against non-compliant companies that miss the required deadlines stipulated for specific product classes during this period [11].

NHP companies are also required to meet good manufacturing practice standards [12] and should have applied for a site license [13] by December 31, 2006. Only manufacturers, packagers, labellers and importers that meet these standards will be allowed to continue to operate in Canada.

In this article, we discuss the perceptions held by leaders of four CAM groups regarding how the implementation of these regulations may impact their practices. Our interviews took place in the fall of 2004 when very little had actually changed in terms of practice or access in the Canadian NHP market place. Therefore, in this article, CAM leaders' perceptions of what *might *happen because of the new NHP regulations are outlined (as opposed to what has actually happened or is actually happening).

### The CAM practitioner groups

Four CAM practitioner groups are included in the current study. These practitioner groups were chosen because NHPs are a core component of their practises in Canada. Although many other practitioners may recommend some types of NHPs (e.g., chiropractors), our focus was on those practitioner groups whose scope of practise is largely dependent on their ability to use NHPs.

Canadian naturopathic practitioners use a combination of clinical nutrition, botanical (herbal) medicine, homeopathic medicine, physical treatments, acupuncture, Asian medicine and lifestyle counselling to stimulate the body's own self-healing abilities [14]. Naturopathic medicine is regulated in four Canadian provinces. Traditional Chinese medicine (TCM) includes a vast range of practices such as acupuncture, herbal medicine, tuina massage and diet therapy [15]. The regulation of TCM varies considerably across Canada. The three fundamental practices of Canadian Western herbalism are: (i) the use of whole plants to treat and prevent illness; (ii) the combination of herbs and (iii) the individualization of herbal combinations in treatment [15,16]. Western herbalists employ a combination of North American and European herbs [16]. Western herbalism is not regulated in any Canadian province. The practice of homeopathy is based on three fundamental principles: the laws of similars (like cures like), individuality (examine each patient's symptoms, physical/mental/emotional, individually) and the use of infinitesimal doses of remedies (the more dilute a remedy the more potent it is) [17,18]. Canadian homeopathic practitioners are also not regulated in any Canadian province.

The new regulations for NHPs represent an emergent instrument that has the potential to (re)shape the behaviour of several groups who have an interest in NHPs. This includes the various practitioner groups, described above, who may previously have had greater freedom in their uses of NHPs in professional practice; and consumers, who may perceive that they now have more open access to NHPs, *because *there is more information on the over-the counter labels. Further, the new Regulations have the potential to create new norms of behaviour. That is, although policies may be initially imposed to change previous practices and behaviours, new practices are eventually internalized as norms that influence people to think, feel and act in particular ways [19,20]. The practitioner groups who include NHPs as an integral part of their professional health practice are likely to be impacted by these new orientations and practices related to NHPs. In this study, we describe these groups' perceptions of how the new NHP regulations are likely to affect their practice, and their place in providing health care alternatives to Canadians.

## Methods

### Design

The design used in this qualitative study is an applied ethnography [21,22]. An applied ethnography is a qualitative research approach that seeks to interpret the meanings of beliefs and behaviours in the context of social/cultural worlds in which individuals are immersed. While a traditional ethnography is focused on describing and interpreting a cultural or social group or system, an applied ethnography is focused on a research population that is defined by some larger social problem, and seeks to describe and expose fields of experience in which meaning is routinely contested. CAM practitioners' role in the Canadian health care system is currently contested. Most CAM definitions describe CAM as those practices that are not part of the dominant health care system. Thus the regulation of NHPs, which are a significant part of the work of many CAM practitioners, is likely to have a significant impact on CAM practices, potentially resulting in the "mainstreaming" of some CAM practitioners. For example, CAM practitioners rely on NHPs (now regulated as therapeutic products) similar to medical doctors who rely on pharmaceutical products. Or, given the new requirements that NHPs are safe for OTC use, these groups may feel their role in advising the public about the safe and appropriate use of NHPs will be usurped.

### Data collection

We conducted key informant interviews [22] with formal and informal leaders of CAM groups in Canada: Western herbalism (n = 9), naturopathy (n = 10), traditional Chinese medicine (n = 8), and homeopathy (n = 10). Formal leaders (e.g. president, chair, head or director) were purposively selected from a list associations, schools and journals to ensure the inclusion of a range of leaders who reflect different segments of each group. Informal leaders were identified by other key informants as part of a "snowball" sampling technique [22]. At the start of the interview, written and informed consent was obtained from each participant. Interviews continued until it became apparent that saturation of the key themes was reached [22]. See Appendix 1 for a copy of the interview guide. This study received ethics approval from the Office of the Vice-President, Research and Associate Provost, Ethics Review Office at the University of Toronto.

The study participants had been practising in Canada from 5 to 35 years. Some participants had formal CAM training from schools in Canada, Europe or China, while others received training in the form of an apprenticeship. All 37 CAM leaders when contacted agreed to participate in the study, with the exception of 1 TCM leader who had retired and 1 homeopathic leader who felt he was not well versed in the regulations to participate in an interview. The TCM leader did however, recommend that one of his colleagues be contacted, and this informant was later interviewed. Based on the overlap in recommendations we had for participants, we are confidant that we were successful in recruiting key CAM leaders whose opinions reflect the four groups' professional opinions about the new regulations.

### Analysis

In order to explore CAM leaders' perceptions of the NHP regulations, interpretative content analysis was used to evaluate their responses to the semi-structured interview questions [23]. The analysis involved identifying themes, sub-themes, and relationships among these. All transcriptions of the semi-structured interviews were entered into Nvivo, a qualitative computer software program [24], used to manage text-data and facilitate our analysis. Each transcript was coded independently by at least two investigators. Interview transcripts were divided into simple single-topic themes, called child-themes. Where relevant, child-themes were placed in a topic category or parent-theme, based on its content and an evident relationship between the parent- and child-theme [22].

## Results

### Naturopathic leaders' concerns about the NHP regulations

In general, naturopathic leaders viewed the new NHP regulations in a positive light. They explained that the public will have more information to make good decisions about self-selected products and that they can be confident that NHPs are meeting specific quality standards. However, the participants expressed some concerns relating to patient safety. They argued the public should not self-diagnose and self-prescribe for certain conditions and that, without practitioner guidance, the use of some herbs may put patients at risk. The cardiac herb, hawthorn, is a good example of this: "As soon as you take hawthorn, you have to assume that there is a cardiac problem and they should be consulting somebody".

Related to this, the naturopath participants feared that high-risk NHPs would be taken off the market and placed on prescription schedules (meaning that CAM practitioners would no longer have access to those NHPs in practice). Many explained that naturopaths should have access to a list or schedule of NHPs that were considered high-risk:

"we were hoping there would be some way by which we could prescribe but that sort of fell through....we were hoping that there would be a separate category just for us or just for providers like us".

Even though the participants would like access to a list of high-risk products, they understood that trying to determine who would have access to "prescribe" or "recommend" these products to patients would be difficult to determine since most CAM practitioners are either not regulated at all in Canada or are not regulated consistently across all provinces:

"It is hard to find a place for our practice given that there is not consistency across the provinces with regulation of naturopathic doctors...so, it is difficult when you are dealing with a federal regulation: how does it translate into your practice in a province where naturopaths are regulated [i.e., Ontario] and in other provinces where they are not?"

Naturopaths were very inclusive in their ideas about who might have the right to access products on this hypothetical restricted schedule. Only one of the ten naturopathic leaders we interviewed argued that no one else besides naturopaths should have such access. The naturopaths believed any health care practitioner providing evidence of appropriate training should have access to these high-risk products. For example, the naturopaths agreed that it would be unfair to restrict qualified TCM practitioners' access to TCM NHPs. However, the naturopaths acknowledged that as long as TCM practitioners remain unregulated in most Canadian provinces, it would be difficult to determine what constitutes a qualified TCM practitioner.

### Western herbalist leaders' concerns about the NHP regulations

In contrast to the naturopathic leaders, Western herbalist leaders were not concerned that the NHP regulations would increase the rate of, or risks associated with, self-medication. One participant explained that herbalists want the public to be able to look after themselves:

"I like the idea that if it was a cold or an insect bite that people should be able to know what to do and we have lost a lot of that....sometimes people need to know how to use things....we have to be able to explain on the label clearly what this is known to be good for and the proper usage of it".

"it's making people, the public more aware of the need for consultation with health professionals. By making, say, that claim on the bottle, it's letting people know that simply buying an over-the-counter remedy, it's not necessarily enough. So I think it's heightening awareness somewhat in the public's eye...OTCs may not be the best choice, or alone aren't the best choice, so it's sending them a little further along the line potentially to the doorstep of a natural health practitioner."

Another participant predicted that the regulations will either have no impact on self-medication or the regulations might even encourage people not to self-medicate: "it may actually reduce self-medication when they start to see all of the contraindications and warnings on labels....the regulations will probably have no effect or possibly reduce people's self-medicating". One participant specified that for serious conditions (i.e., cancer) the public should be encouraged to consult an appropriate practitioner, whether conventional or complementary.

Like the naturopaths, several herbalist leaders thought it would be beneficial if herbalists had access to some high-risk products that were not directly accessible to the public. One herbalist practitioner made it clear that if there was such a schedule, it should only be accessible to registered herbalists, and not only to pharmacists, as is the case currently for some drugs. The herbalists were less clear about which other CAM practitioner groups should have access. Western herbalist leaders realized that their unregulated status would make it difficult to determine if they might be qualified to prescribe NHPs. However, Western herbalists specifically indicated that they did not see pharmacists as appropriate gate-keepers of NHPs, given their lack of knowledge about herbs.

### Traditional Chinese medicine leaders' concerns about the NHP regulations

TCM leaders did not think that the NHP regulations would increase or discourage self-medication use among the public. According to one TCM leader:

"there may be less of a tendency for people to go and self-treat, unless they are Chinese and unless it has been part of their tradition and culture....I don't think many people would walk in and just buy something off the shelf. It depends on how they are going to be sold and where they are going to be allowed to be sold".

Many TCM leaders were concerned about how NHPs, typically used by TCM practitioners, would be labelled. Some herbs or NHPs may have both Western and TCM uses. There was some uncertainty about whether or not both TCM and Western uses of an herb will be labelled on NHPs. TCM leaders were concerned that their unique knowledge will be ignored if only the Western use of the herb is described on the label. However, if the TCM uses were listed on the bottle they might be taken out of the context of the TCM philosophy which these leaders described as problematic: "for example, if the label just says headaches, but it may not treat all headaches, you know, it might treat only one particular kind of headache".

TCM leaders also feared that the Natural Health Products Directorate (NHPD) may over-regulate NHPs, taking some high-risk products off the market. Like the other leaders, they suggested a restricted schedule of high-risk NHPs, similar to conventional drugs:

"I think that there are some herbs that could be deemed, you know, toxic or dangerous or should only be prescribed by a practitioner and dispensed by a pharmacy....that would protect the public and give practitioners access to that particular product".

Like the naturopaths and the Western herbalists, determining who should have access to those products was identified by the TCM leaders as potentially very complicated. However, the TCM leaders described that only TCM practitioners should have access to high-risk TCM NHPs, because they are the only qualified practitioners to handle these high-risk products: "naturopaths have also done a little bit of traditional Chinese medicine... it seems unfair that they should be allowed to prescribe something that they don't have extensive knowledge in".

### Homeopathy leaders' concerns about the NHP regulations

Similar to the TCM leaders, the self-medication issues were of no concern for homeopathic leaders:

"I think most people realize that certain things should not be used without consulting a practitioner, because of the toxicity of the products. For example, [regarding] Belladonna, the label would explain what would happen, what they're dealing with...so I don't think it [self medication] will be much of an issue.

Although they did not advocate a "restricted schedule" of products like the other practitioners, the homeopathic leaders were concerned about maintaining access to homeopathic products. This concern was related to the homeopaths' views that personnel within the NHPD may not be sufficiently competent to deal with homeopathic products. For example, three homeopathic leaders described what they perceived as the lack of homeopathic experience and homeopathic education among members of the NHPD. There were numerous references to NHPD members' lack of understanding of the principles of homeopathy. The homeopathic leaders explained that during the development of the regulations, they had to continuously reiterate these concerns to the members of the NHPD. One participant explained that the individuals with homeopathy expertise working at the NHPD do not in his opinion have sufficient education and experience in homeopathy.

## Discussion

All CAM practitioners were concerned with issues of their own access to NHPs, but specific concerns differed between groups. For example, all CAM groups included in the study, with the exception of the homeopathic leaders, specifically indicated a desire to have a restricted schedule of NHPs that would be available to CAM practitioners and secondly, the naturopathic leaders were the only group concerned that the NHP regulations could potentially endanger patients if they self-medicate incorrectly with NHPs because of unrestricted access to some NHPs.

Access issues dominated all the interviews. A restricted schedule of NHPs that would be available to CAM practitioners was discussed by many as a possible way to protect practitioners' access to NHPs, which are necessary for their practises even if some NHPs are no longer available to the general public. The naturopaths expressed a desire for a restricted list of NHPs made available to themselves and other qualified practitioners. Naturopaths are regulated in four Canadian provinces and having the power to "prescribe" from such a list could be seen as a way to legitimize their knowledge claims. TCM leaders also hoped to have access to high-risk NHPs and some even argued that if they are successful in becoming regulated they would have access to those products even if they were in a restricted schedule. Western herbalists saw great value in having such a list, but worried that, because of their unregulated status, they may not have access to it and homeopathic practitioners did not identify such a list as important.

There were conflicting views regarding who should have access to high-risk products, and where they should be available. Based on our findings, CAM practitioners viewed themselves as the legitimate gatekeepers of NHPs (based on their unique knowledge base) and they explained that conventional practitioners' access to these products should be limited. It would be interesting to determine if physicians, who are the gatekeepers of prescription medications, would agree with this. Another important consideration is that some drugs in Canada are restricted to sales in pharmacies (i.e., the patient must access the drug through a pharmacist). If certain NHPs were to be restricted to sale in pharmacies, the public would have to consult a pharmacist to receive them. No CAM group suggested this as a viable option.

The naturopathic leaders were the most inclusive in regards to which practitioners should have access to any restricted schedule of NHPs. Their perspective could be based on their education (i.e., they learn from a range of philosophies including Western herbalism, TCM, homeopathy, manipulation, nutrition etc.). The naturopathic practitioners' inclusiveness may also be a result of the fact that they felt more involved in, and in control of, the major components of the development and implementation of the NHP Regulations because naturopaths had greater representation on the NHPD than any other CAM practitioner group (i.e., the Director General of the NHPD is a naturopathic practitioner). This may have given the naturopaths confidence that the implementation of the NHP regulations will not threaten their core practices, and indeed, may have allowed the leaders of naturopathy to focus on details that may strengthen and further legitimize their role in Canadian health care.

Naturopaths were the only group that expressed a concern that once the new regulations are fully implemented, members of the public may no longer feel the need to consult either a naturopath or any other practitioner. This appeared to be indicative of a concern about restricted access and professional "turf", based on naturopaths' claims to having specific knowledge of, and expertise around the uses of NHPs. The regulation of NHPs as over-the-counter products appears to suggest disregard for naturopath's claims to knowledge and its unique approaches to health promotion or treatment of illness. It is notable that the naturopath leaders' concerns about "off label" use, or inappropriate use of NHPs were similar to the conventional literature on OTC use where it has been argued that selecting the appropriate OTC medication can be very confusing (and potentially harmful) for consumers, particularly because of numerous emergent "line extensions", presented as "new" products [25].

In contrast to the concerns of naturopaths, Western herbalist leaders were not concerned that the NHP regulations would encourage self-medication use and this could potentially be related to their philosophy of care and practice. Western herbalists reported encouraging their patients to learn to heal by themselves without the help of a practitioner.

Both TCM and homeopathy leaders did not identify changes in self-medication behaviour as a potential outcome of the new regulations. It appeared that neither group viewed self-medication as a threat to their practice. This may be because the complex theories that govern TCM and homeopathy product choice make these products less accessible to the general public. All but a few "first aid" remedies require the practitioner's guidance for effective use. TCM and homeopathic products were previously available directly to consumers, but the pubic lacked the expertise to self-select in most cases. TCM and homeopathic leaders did not identify the new information required on NHP labels as a threat to their expert knowledge. However, TCM leaders were concerned that TCM NHPs may be labelled inappropriately in that they would not accurately reflect TCM philosophy.

The findings suggested that these groups' diverse practices are not usefully combined into a single entity called CAM, which is defined in comparison to the dominant medical system. There was diversity between the different CAM groups, just as there is between "CAM" and conventional medicine. In fact, one of the reasons it was so difficult to define CAM is the lack of uniform underlying beliefs, principles or practices that are common to all CAM practitioner groups. For example, the Western herbalists, homeopaths and TCM leaders that we spoke to were concerned about the fact that a naturopath was the head of the Directorate and that their specific practice issues may not be addressed. Just because these groups differed significantly from conventional medicine, does not mean their interests were the same. Although the naturopaths, homeopaths, Western herbalists and TCM practitioners practice largely outside the conventional health care system, they were all fighting for space within that system. Policy makers should not ignore the nuances of that struggle.

This is an exploratory qualitative study in which we provided an in-depth examination of CAM leaders' perceptions of the NHP regulations and the perceived impact on their professional groups. We spoke to the key naturopathic, homeopathic, Western herbalist and TCM leaders in Ontario and we believe the findings are an accurate reflection of their opinions but may not be generalizable to leaders in other jurisdictions or to other "CAM" providers besides the four groups in this study. Finally, there is no official list of Canadian CAM associations and schools. Therefore, some CAM associations and schools may not have been included in the list of CAM associations and schools that was developed prior to data collection. However, a snowball sampling technique was employed, which allowed us to capture some associations, and schools that were initially missed. Interviews continued until key themes were saturated.

As the regulations continue to be implemented in Canada, it will be important to explore the actual impact of these regulations on the naturopathic, homeopathic, Western herbalist and TCM practices that were the focus of this study. Of particular relevance will be whether or not these groups actually lose access to particular NHPs and if so how they handle the loss of products, and what impact it has on their practice or access to products. Rank and file practitioner opinions of the NHP regulations will also be important to research because they may differ significantly from the opinions of the leaders, given at times, the lack of cohesion among these groups, as described by Welsh et al.[26].

## Conclusion

Naturopaths, TCM practitioners, homeopaths, and Western herbalists were all concerned about how the new NHP regulations will affect their access to the products, which they need to practice effectively. The new regulations define NHPs as over the counter products, thus CAM leaders, particularly the naturopathic leaders, were concerned that their role as health care providers will be limited.

## Competing interests

The author(s) declare that they have no competing interests.

## Authors' contributions

KM: made substantial contributions to the design of the study, the collection of the data as well as the interpretation and analysis of the data. KM also drafted the manuscript and gave final approval of the version to be published.

HB: made substantial contributions to the design of the study as well as the interpretation and analysis of data. HB was involved in revising the manuscript critically and for important intellectual content. HB also gave final approval of the version to be published.

PB: made contributions to the design of the study as well as the interpretation of the data. PB was involved in revising the manuscript critically and for important intellectual content. PB also gave final approval of the version to be published.

NK: Made substantial contributions to the collection of data as well as the interpretation and analysis of the data. NK was involved in revising the manuscript and gave final approval of the version to be published.

## APPENDIX 1

### Semi-structured interview questions

1. How did you become interested in *naturopathy, homeopathy, traditional Chinese medicine or Western herbalism *and what are some of the guiding principles that inform your practice?

2. What do you know or what have you heard about the new NHP regulations?

#### Probes

a) Why do you feel the new NHP regulations were developed?

b) Where you consulted in the development of the new NHP regulations by the Natural Health Product Directorate?

c) Have you attended any information sessions or seminars on the new regulations?

d) Have you found information on the new NHP regulations to be readily available?

3. What do you think about the information you have received regarding the new regulations?

4. How do you feel these regulations will impact the goals, practices and philosophical traditions of *homeopathy? naturopathy? traditional Chinese medicine? or Western herbal medicine?*

#### Probes

a) What are your thoughts regarding the statement that "it appears that CAM practitioners who manufacture large batches of NHPs at one time to treat numerous patients will have to apply for site licenses and product licenses under the new regulations".

b) What are your thoughts about whether it should be stated on the label of certain NHPs that a *homeopathic, naturopathic, traditional Chinese medicine or Western herbal medicine *practitioner should be consulted when taking this product?

c) In regards to the current list of NHPs listed on Schedule 1 of the new regulations, do you feel there should be other NHPs included on Schedule 1 that are currently regulated under other Acts?

d) What are your feeling surrounding the possibility that *homeopathic, naturopathic, traditional Chinese medicine or Western herbal medicine *practitioners will loose access to certain NHPs as a result of the new regulations?

e) Homeopathic Leaders: What are your feelings surrounding the fact that homeopathic products will keep their 8-digit DIN number as oppose to transferring to a natural product number (NPN)?

f) Naturopathic Leaders: How do you feel about the inclusion of NHPs in the proposed scope of practice stated in the 2001 HPRAC report?

5. How do you foresee these regulations impacting the ability of *homeopathic, naturopathic, traditional Chinese medicine or Western herbal medicine *practitioners to practice in a manner consistent with their philosophical traditions?

6. According to the government, the NHP regulations are suppose to bring improved access to safe NHPs, while respecting Canadian's freedom of choice and cultural diversity. What are your thoughts about this statement?

version date (05/04/04)

## Pre-publication history

The pre-publication history for this paper can be accessed here:


